# Analyses of density-dependent effects are needed to understand how and when *Wolbachia* can control dengue vectors

**DOI:** 10.1186/s12915-016-0328-4

**Published:** 2016-11-18

**Authors:** Robert A. Cheke

**Affiliations:** Natural Resources Institute, University of Greenwich at Medway, Central Avenue, Chatham Maritime, Chatham, Kent ME4 4TB UK

## Abstract

Releases of *Wolbachia*-infected mosquitoes have been shown to be an effective method of controlling *Aedes aegypti*, the main vector of dengue fever, in Australia. A study in *BMC Biology* from Penelope Hancock and others shows that incorporation of density-dependent effects into population models can provide major improvements in understanding how and when the infected populations can become established.

See research article: https://bmcbiol.biomedcentral.com/articles/10.1186/s12915-016-0319-5.

## Commentary

**Fig. 1:**
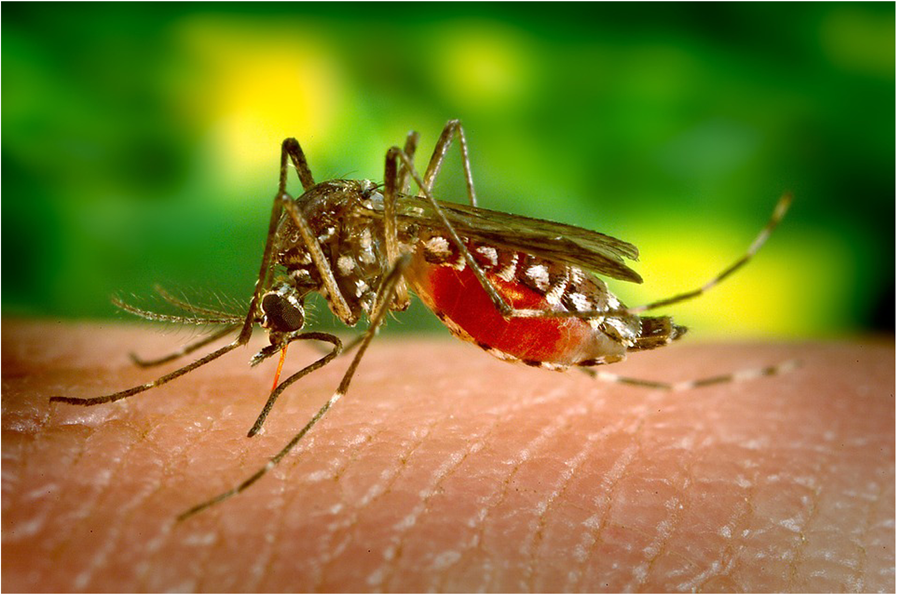
An *Aedes aegypti* mosquito drawing blood from a human. These organisms can carry dengue, Zika and Chikungunya viruses and are primarily responsible for the person-to-person transmission of these viruses through bites during feeding

As many as 390 million people are estimated to be infected with the dengue virus [1] and increasing numbers by the Zika and Chikungunya viruses, all three of which are mainly transmitted from person to person by the mosquito *Aedes aegypti* (Fig. 1). Traditionally, the spread of dengue was combatted by minimising the mosquitoes’ breeding opportunities through covering or treating water receptacles such as storage pots to prevent adults from laying eggs or emerging from pupae and/or by spraying insecticides to kill the adults, principally in urban areas. Concentrations of people in conurbations where breeding opportunities for the vectors are legion are ideal conditions for this disease to spread. Dengue has also benefitted from population growth, poor urban infrastructure and networks of international travellers. Fortunately, there is now a new weapon in the armoury of entomologists’ killing agents which seems too good to be true as it is self-perpetuating and apparently environmentally benign. This ‘silver bullet’ exploits the presence in many insects of endosymbionts in the shape of *Wolbachia* bacteria, but these bacteria can be double-edged swords as some confer advantages to their hosts and others are disadvantageous to them.

The *Wolbachia* that can infect *Ae. aegypti*, which are not normally infected in the wild, have been exploited to control the vectors since the bacteria reduce the ability of the mosquitoes to transmit the viruses that they carry, enhance the fitness of infected hosts and at the same time prevent uninfected hosts from breeding owing to a phenomenon known as cytoplasmic incompatibility (CI). This occurs when an infected male mates with an uninfected female and then the female is unable to produce viable offspring, but it is not yet understood how this is achieved. The bacteria are maternally transmitted, so when an infected pair mate or when an infected female mates with an uninfected male the *Wolbachia* infection propagates with the insects, but not when uninfected females copulate with infected males as then the CI will lead to the death of the mosquito embryos. Thus, the introduction of infected populations into uninfected ones should lead to increases in the proportion of infected individuals to the detriment of the uninfected proportion. Because the infected mosquitoes are less able or unable to spread the viruses, transmission of the disease becomes interrupted. That is precisely what has been achieved in Australia [2], but elsewhere the method has sometimes failed. Hancock et al. [3] have now provided an explanation for the discrepancy between outcomes of different control programs by elegantly combining results of experiments with mathematical modelling. Their research adds substantially to the understanding of both the theory and practice of the nascent science of using *Wolbachia* to control disease vectors.

The theory of introductions of *Wolbachia*-infected insects is analogous to that for introductions using the sterile insect technique (SIT) originally devised by Knipling [4] and used in practice against screwworms, tephritids and moths [5]. But, as with all simple ideas and models, reality brings new complications. For the mosquitoes to be controlled by introducing *Wolbachia*-infected insects, there are tactics based on whether the targets should be suppressed or eliminated and careful consideration is needed not only of the ratio of introduced *Wolbachia*-infected to uninfected wild insects but also in what proportions the two sexes should be released and how often. In addition, there are different strains of the bacteria, each with their own properties.

The successful releases in Australia in 2011 used populations infected with the *WMel* strain of *Wolbachia*, which has strong anti-dengue abilities and confers low fitness costs. Use of the same strain was also successful in Indonesia in 2014 but introductions with the *wMelPop* strain, which reduces its hosts’ longevity, in Vietnam in 2013 failed. So, before large scale releases are made in Brazil to fight the Zika and dengue epidemics there [6], reasons accounting for successes or failures are needed. Conclusions from research will also assist releases planned or ongoing in China, Colombia, Malaysia and Singapore and will need to address how to succeed in urban as opposed to rural settings.

Because many characteristics of animal and plant populations vary with their densities, Hancock et al. assumed that the same would be the case with *Ae. aegypti*, particularly for their larvae. Therefore, they experimented with two populations in field cages. One population was started as an uninfected group, which was then regularly seeded with *WMel*-*Wolbachia*-infected individuals after two months. A second population was initiated with 40% *WMel*-*Wolbachia*-infected adults and left to its own devices. In both cases the times taken for the larvae to develop into pupae were longer at higher larval densities; in other words the development times were density-dependent. This was by and large true for both infected and uninfected individuals, as was a density-dependent decline in the females’ fecundities. Quantitative relationships for these effects were then included in population models, which were elaborated to mimic introductions of a fixed number of *WMel*-*Wolbachia*-infected adults being released into an uninfected population every week during a three-month period. The overall number to be released is calculated on the basis of the ‘release ratio’—the size of each release divided by the initial wild population size. Next, estimates were made of the minimum release ratio needed to achieve a 60% establishment of infected individuals one week after the final release, followed by the time taken after this to establish a 95% infection rate.

Despite uncertainties regarding mortality rates and relative fitness with regard to, for example, insecticide susceptibility, Hancock et al. were able to show that the eventual outcome depended heavily on local density-dependent effects such as the relative fitnesses of the introduced and targeted (wild) mosquito populations at the release sites. Indeed, the ratio of released to wild mosquitoes needed to attain the required percentage of infections could vary by an order of magnitude and the time taken for this aim to be achieved might vary by as much as two years. This is of particular significance as in field trials it has been noted that some establishments of infected populations differ in the times taken to achieve adequate ratios of infected to uninfected or have declined after initial successes.

Hancock et al.’s research has shown the importance of incorporating laboratory and field data into mathematical models and that explicit recognition of density-dependent mechanisms has important practical implications. The publication is timely as the Wellcome Trust and the Bill and Melinda Gates Foundation, together with UK and Brazilian Governments, are about to embark on a US$18 million scheme to use *Wolbachia* against *Ae. aegypti* in Brazil [6]. However, whilst a major boost to improve planning, I would argue that Hancock et al.’s models are not quite a panacea as the story also needs to take account of the transmission success of *Wolbachia* going from female mosquitoes to their offspring, which may not always be perfect [7]; the sex ratios of released populations [8]; and the lengths of intervals between release timings [8]. Then there is the Asian tiger mosquito, *Ae. albopictus*, another major vector of dengue that occurs in Brazil and is spreading fast in many parts of the world, including Europe, which can also be targeted with *Wolbachia* [9].
